# Optimized End Functionality of Silane-Terminated Liquid Butadiene Rubber for Silica-Filled Rubber Compounds

**DOI:** 10.3390/polym15122583

**Published:** 2023-06-06

**Authors:** Sanghoon Song, Haeun Choi, Junhwan Jeong, Seongyoon Kim, Myeonghee Kwon, Minji Kim, Donghyuk Kim, Heungbae Jeon, Hyun-jong Paik, Sungwook Chung, Wonho Kim

**Affiliations:** 1 School of Chemical Engineering, Pusan National University, Busan 46241, Republic of Korea; thdtkd1111@gmail.com; 2 Department of Polymer Science and Engineering, Pusan National University, Busan 46241, Republic of Korea; fexox06112@gmail.com (H.C.); kimsy9780@gmail.com (S.K.); 3 School of Chemical and Biomolecular Engineering, Pusan National University, Busan 46241, Republic of Korea; wnsghks4192@gmail.com; 4 Department of Chemistry, Kwangwoon University, Seoul 139-701, Republic of Korea; myeonghee814@gmail.com (M.K.); annie73329@gmail.com (M.K.); 5 Hankook Tire & Technology Co., Ltd., R&D Center, 50 Yuseong-daero 935beon-gil, Daejeon 34127, Republic of Korea; ehdgurzxc@gmail.com

**Keywords:** liquid butadiene rubber, radical polymerization, silica-filled compound, rubber compounding, passenger car radial tire tread

## Abstract

As the world is shifting from internal combustion engine vehicles to electric vehicles in response to environmental pollution, the tire industry has been conducting research on tire performance to meet the requirements of electric vehicles. In this experiment, functionalized liquid butadiene rubber (F-LqBR) with triethoxysilyl groups at both ends was introduced into a silica-filled rubber compound as a substitute for treated distillate aromatic extract (TDAE) oil, and comparative evaluation was conducted according to the number of triethoxysilyl groups. The results showed that F-LqBRs improved silica dispersion in the rubber matrix through the formation of chemical bonds between silanol groups and the base rubber, and reduced rolling resistance by limiting chain end mobility and improving filler–rubber interaction. However, when the number of triethoxysilyl groups in F-LqBR was increased from two to four, self-condensation increased, the reactivity of the silanol groups decreased, and the improvement of properties was reduced. As a result, the optimized end functionality of triethoxysilyl groups for F-LqBR in silica-filled rubber compound was two. The 2-Azo-LqBR with the optimized functionality showed an improvement of 10% in rolling resistance, 16% in snow traction, and 17% in abrasion resistance when 10 phr of TDAE oil was substituted.

## 1. Introduction

In response to greenhouse gas emissions and various environmental pollution problems around the world, interest in sustainability and eco-friendly transportation, for which the power source does not emit pollutants, is growing [1,2]. Accordingly, the automotive industry has focused on the transition from internal combustion engine vehicles to electric vehicles [3,4], and the tire industry is researching tire designs that meet the performance requirements of electric vehicles. Because of the nature of the electric power source, electric vehicles apply high torque to the tires from the beginning of acceleration, and the high battery load requires a better wear resistance for the tires [5,6]. In addition, because of the capacity limitations of the batteries, a dramatic reduction in rolling resistance is required to ensure a sufficiently long driving range [5,7]. Therefore, tire manufacturers are actively trying to apply new materials to improve the performance of tires for electric vehicles [8]. 

Tire tread is the most crucial element in determining tire performance [9] as it contributes the largest volume fraction [10], protects the carcass and belt layers, and is in direct contact with the road surface. In passenger car radial tires, a blend of solution styrene-butadiene rubber (SSBR) and butadiene rubber (BR) is used as the base rubber [11,12]. To achieve better traction and rolling resistance, conventional carbon black fillers are being replaced with silica/silane systems [13,14]. To enhance the compound’s processability, manufacturers add processing aids like TDAE oil [15]. However, adding processing oil is unfavorable for the abrasion resistance and rolling resistance of the compound [16,17]. Additionally, when the tire operates for a long time, a migration problem occurs in which processing oil migrates to the tire surface. This, in turn, reduces the suppleness of the tire tread and its physical properties [18]. Therefore, the demand for liquid butadiene rubber (LqBR) as an alternative to processing oils has begun to grow.

LqBR has a higher molecular weight (1000 to 50,000 g/mol) than processing oils. Due to the presence of double bonds, LqBR acts as a co-vulcanizable plasticizer that can be crosslinked with base rubber [19]. Thus, LqBR shows less migration compared to processing oils, which provides stable long-term performance over extended driving times [20]. Furthermore, by controlling the microstructure of LqBR, the compound’s glass transition temperature (T_g_) can be regulated. The addition of LqBR with a low T_g_ reduces the T_g_ of the compound, enhancing its abrasion resistance [21].

Kuraray Co., Ltd. (Tokyo, Japan) confirmed that the co-vulcanization of non-functionalized LqBR (N-LqBR) with base rubber is possible through toluene extraction experiments [22]. It was also confirmed that the viscoelastic properties in the low-temperature region and abrasion resistance were concurrently enhanced. Continental AG in Germany applied N-LqBR to enhance the abrasion resistance while maintaining the wet traction of tire tread [23]. In contrast, Kitamura et al. reported a deterioration in fuel efficiency due to hysteresis loss, resulting from the free chain ends of LqBR when N-LqBR was applied into the compound [24]. This implies the need to introduce functional groups to limit the chain-end mobility of LqBR. 

Responding to the need for F-LqBR, Cray Valley Co., Ltd. (Exton, PA, USA) and Evonik Industries AG (Essen, Germany) have conducted studies on silane-functionalized LqBR and successfully commercialized them [25,26], and the tire industry has reported studies on its application in rubber compounds [27,28,29]. Kim et al. synthesized F-LqBRs with different functional group positions and applied them to silica-filled rubber compounds to determine the effects of the functional group positions and free chain ends of LqBR on the properties of tire tread compounds [30]. As a result, the functional group of F-LqBRs were bonded on the silica surface, so there was no free chain end effect. Thus, it was confirmed that the addition of F-LqBR, which functionalizes at both ends of the chain, can concurrently enhance the fuel efficiency and abrasion resistance of a compound. 

Prior studies have investigated the applicability of LqBR and the effects of functionalization and the macro- and microstructure of the polymer and verified the superiority of both chain-end functionalized LqBR. However, since the synthesis of telechelic polymers requires advanced technics related to initiator design and molecular weight control [31,32], there have been no previous studies on the effect of the number of end-functional groups of F-LqBR on the compound properties. 

Therefore, we designed and synthesized a functional initiator to operate during the polymerization step [33,34], which is one of the methods for synthesizing polymers with a telechelic structure. Considering that the initiation mechanism, initiation efficiency, concentration, and solubility of the initiator can affect polymerization [35,36,37], we designed an initiator structure suitable for the polymerization of 1,3-butadiene. Furthermore, we synthesized F-LqBRs with end functionalities of two or four triethoxysilyl groups. These F-LqBRs were then used as processing aids for preparing silica-filled rubber compounds to evaluate the effect of the number of end-functional groups of LqBR on the compound’s properties. This research provides a basis for the selection of optimized end-functional groups and F-LqBR structures, toward improving both the wear resistance and fuel efficiency of tires required for electric vehicles, while ensuring long-term performance.

## 2. Materials and Methods

### 2.1. Synthesis of the Functional Initiator 

#### 2.1.1. Synthesis of (E)-4,4′-(diazene-1,2-diyl)bis-(4-cyano-N-(3-(triethoxysilyl)propyl)pentanamide) (difunctional initiator) 

4,4′-azobis(4-cyanovaleric acid) (ACVA, 98%, Sigma-Aldrich Corp.; Seoul, Republic of Korea), (3-aminopropyl) triethoxysilane (98%, Sigma-Aldrich Corp.; Seoul, Republic of Korea), and phosphorus pentachloride (98%, SigmaAldrich Corp, Seoul, Republic of Korea) were used as reagents, and dichloromethane (DCM, 99%, Duksan General Chemical Co.; Seoul, Republic of Korea) was used as solvent. For purification, n-hexane (95%, Duksan General Chemical Co.; Seoul, Republic of Korea) and diethyl ether (99%, Daejung Chemicals & Metals Co.; Siheung, Republic of Korea) were used. 

#### 2.1.2. Synthesis of (E)-4,4′-(diazene-1,2-diyl)bis(4-cyano-N,N-bis(3-(triethoxysilyl)propyl)pentanamide) (tetrafunctional initiator) 

ACVA, bis[3(triethoxysilyl)propyl]amine (95%, Gelest Inc.; Morrisville, PA, USA), phosphorus pentachloride (98%, Sigma-Aldrich Corp, Seoul, Republic of Korea), and triethylamine (TEA, 98%, Duksan General Chemical Co.; Seoul, Republic of Korea) were used as reagents, and dichloromethane (DCM, 99%, Duksan General Chemical Co.; Seoul, Republic of Korea) was used as a solvent. N-hexane (95%, Duksan General Chemical Co.; Seoul, Republic of Korea) and diethyl ether (99%, Daejung Chemicals & Metals Co.; Republic of Korea) were used for purification. 

#### 2.1.3. Polymerization 

For polymerization, the reaction mixture was dissolved in tetrahydrofuran (THF, 99.9%, Samchun Chemical Co.; Seoul, Republic of Korea). The functional initiators were prepared as described above. Radical polymerization was performed using 1,3-butadiene (Kumho Petrochemical Co., Ltd.; Daejeon, Republic of Korea), which was used as received without further purification. 

#### 2.1.4. Compounding 

SSBR (SOL-5220M, Kumho Petrochemical Co. Daejeon, Republic of Korea, styrene content: 26.5 wt%, vinyl content: 26 wt%, non-oil extended) and high-cis butadiene rubber (CB24, Lanxess Chemical Industry Co., Ltd.; Cologne, Germany; cis content: 96 wt%) were used as the base rubber. Silica (ZEOSIL 195MP, Solvay Silica Korea Co., Ltd.; Gunsan, Republic of Korea) was used as a filler. The silane coupling agent was X50-S (Evonik Industries AG, Essen, Germany; bis-[3-(triethoxysilyl)propyl]tetrasulfide (TESPT) 50%, carbon black N330 50%). Zinc oxide (Sigma-Aldrich Corp.; Seoul, Republic of Korea) and stearic acid (Sigma-Aldrich Corp.; Seoul, Republic of Korea) were used as crosslinking activators. N-(1,3-dimethybutyl)-N′-phenyl-phenylenediamine (6PPD, Kumho Petrochemical Co., Ltd.; Daejeon, Republic of Korea) and 2,2,4-trimethyl-1,2-dihydroquinoline (TMQ, Sinopec Corp.; Beijing, China) were used as antioxidants. Sulfur (Daejung Chemicals & Metals Co.; Siheung, Republic of Korea) was used as a crosslinking agent, while cyclohexyl benzothiazole-2-sulfenamide (CBS; 98%, Tokyo Chemical Industry Co., Ltd.; Tokyo, Japan) and 1,3-diphenylguanidine (DPG, 98%, Tokyo Chemical Industry Co., Ltd.; Tokyo, Japan) were used as vulcanization accelerators. For processing aids, four different substances were prepared: TDAE oil (Kukdong Oil & Chemicals Co., Yangsan, Republic of Korea) and N-LqBR (LBR-307, Kuraray Co., Ltd.; Tokyo, Japan), which are commercially available, and 2-Azo-LqBR and 4-Azo-LqBR, which were synthesized using functional initiators (as explained in detail in Section 2.4).

### 2.2. Characterization 

#### 2.2.1. Gel Permeation Chromatography (GPC)

GPC (Shimadzu, Kyoto, Japan) was used to determine the number average molecular weight of the synthesized polymer. A solvent delivery system, a refractive index detector, and columns were used for the GPC experiment. The columns used were as follows: a HT 6E (10 µm, 7.8 mm × 300 mm), a HMW 7 column (15–20 µm, 7.8 mm × 300 mm), and a HMW 6E column (15–20 µm, 7.8 mm × 300 mm). Polybutadiene standards (Kit Poly(1,3-butadiene) number average molecular weight (M_n_) standard, WAT035709, Waters Corp.; Eschborn, Germany) were used for molecular weight calibration.

#### 2.2.2. Proton Nuclear Magnetic Resonance Spectroscopy (^1^H NMR)

The molecular structure of LqBR was confirmed by ^1^H NMR spectroscopy (Varian, Unity Plus 300 spectrometer, Garden State Scientific, Morristown, NJ, USA), using deuterochloroform (CDCl_3_, Cambridge Isotope Laboratories, Inc.; Andover, MA, USA) as the solvent. 

#### 2.2.3. Differential Scanning Calorimetry (DSC)

T_g_ was confirmed using a DSC (DSC-Q10, TA Instruments, New Castle, DE, USA). DSC measurements were performed at temperatures ranging between −100 and 100 °C at a heating rate of 10 °C/min. After the first heating-cooling cycle, T_g_ was obtained during the second heating.

#### 2.2.4. Payne Effect 

Strain sweep tests (0.28–40.04%) at 60 °C were performed using a rubber processing analyzer (RPA 2000, Alpha Technologies, Hudson, Ohio, USA) to analyze the degree of filler network formation in the unvulcanized compound. As the strain increases, the filler network is destroyed and the storage modulus (G′) decreases. Thus, ΔG′ (G′ at 0.28% minus G′ at 40.04%) shows the degree of filler-filler interaction, as described by the Payne effect [38]. 

#### 2.2.5. Mooney Viscosity 

A Mooney rotatory viscometer (Vluchem IND Co.; Seoul, Republic of Korea) was used for measuring the Mooney viscosity, which is correlated to the processability of a compound. Measurements were performed using a large rotor (diameter 38.10 ± 0.03 mm, thickness 5.54 ± 0.03 mm) at 100 °C and 2 rpm for 4 min after preheating for 1 min according to the ASTM D1646 conditions. 

#### 2.2.6. Curing Characteristics 

A moving die rheometer (RLR-3-rotorless rheometer, Toyoseiki, Tokyo, Japan) was used for the analysis of the curing characteristics of the compounds at 160 °C for 30 min under an angular displacement of ±1°. In this experiment, the minimum torque (T_min_), maximum torque (T_max_), and optimal cure time (t_90_) were obtained. Then, t_90_ was used for preparing the vulcanizates in a heating press at 160 °C. 

#### 2.2.7. Solvent Extraction and Crosslink Density 

The vulcanizates, with pieces of 10 mm (length) × 10 mm (width) × 2 mm (thickness), were prepared and weighed. The specimen was immersed in THF and n-hexane at 25 °C for 2 days each to eliminate the organic additives. The samples were then dried at 25 °C for 1 day and weighed, and the mass fraction of the extracted organic additives was calculated. The samples were then swollen in toluene at 25 °C for 1 day and weighed again. The crosslink density was calculated from the measured weight of the sample using the Flory–Rehner equation below [39,40,41]:

(1) $$\nu =\frac{1}{2M_{c}}=-\frac{\ln\left(1-V_{1}\right)+V_{1}+\chi V_{1}^{2}}{2\rho_{r}V_{0}\left(V_{1}^{\frac{1}{3}}-\frac{V_{1}}{2}\right)}$$

where *ν* is the crosslink density of vulcanizates (mol/g), *M*_C_ is the average molecular weight between crosslink points (g/mol), *V*_1_ is the volume fraction of rubber in the swollen gel at equilibrium, *V*_0_ is the molar volume of solvent (cm^3^/mol), *ρ*_r_ is the density of the rubber (g/cm^3^), and *χ* is the polymer–solvent interaction parameter. 

#### 2.2.8. Mechanical Properties 

The properties of dumbbell-shaped specimens of 100 mm (length) × 25 mm (width) made according to ASTM D 412 were estimated using a universal testing machine (UTM, KSU-05M-C, KSU Co., Ansan, Republic of Korea). The tensile test for evaluating the mechanical properties of the vulcanizates was conducted at 500 mm/min and repeated three times, and the median value was used according to the ASTM standard. 

#### 2.2.9. Abrasion Resistance 

The abrasion resistance test was conducted using a cylindrical sample with diameters of 16 mm and thicknesses of 8 mm. First, the initial mass was measured, and the specimen was ground for 40 s at a speed of 40 rpm under a load of 5 N using a Deutsche Industrie Normen (DIN) abrasion tester. The mass loss was then calculated by measuring the mass of the specimen. 

#### 2.2.10. Dynamic Viscoelastic Properties 

The dynamic viscoelastic properties were obtained using a dynamic mechanical analyzer (DMA; Q800, TA Instrument, New Castle, DE, USA) and a dynamic mechanical thermal spectrometer (DMTS; EPLEXOR 500N, GABO Instruments GmbH, Ahlden, Germany). First, the T_g_ and dynamic viscoelastic properties of the vulcanizates were measured over a temperature range of −80 °C to 80 °C using DMA. Then, the DMTS was used to measure tan δ through strain sweeps from 0.5% to 10% in tension mode at a temperature of 60 °C and a frequency of 10 Hz. 

### 2.3. Synthesis of the Functionalized Initiator 

#### 2.3.1. Di-functional Initiator (2-Azo-initiator) 

**Scheme 1:**
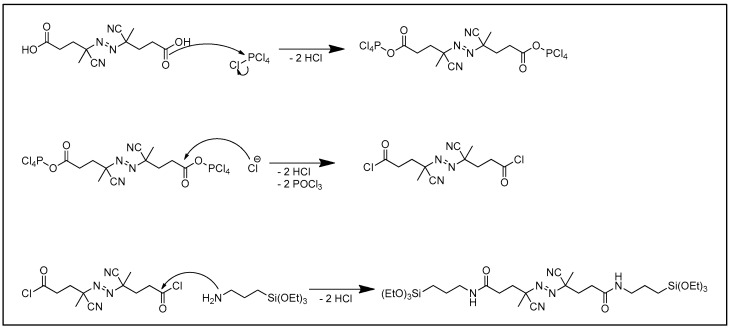
Synthesis of difunctional initiator.

The synthesis of the di-functional initiator substituted on both sides by silane is shown in Scheme 1. One equivalent of ACVA (5 g, 17.8 mmol) was dissolved in 150 mL of dichloromethane under an Ar atmosphere at 25 °C, and then cooled to 0 °C. Then, 2.5 equivalents of phosphorus pentachloride (9.3 g, 44.6 mmol) were added for 30 min and then stirred at 25 °C for 1 h. A measure of 120 mL of dichloromethane was removed using vacuum distillation, 70 mL of hexane was added, and recrystallization at 0 °C produced €-′,4′-(diazene-1,2-diyl)bis(4-cyanopentanoyl chloride) as a white solid. Then, 1 equivalent (4.5 g, 14.2 mmol) of the previously obtained €(′)-4,4′(diazene-1,2-diyl)bis(4-cyanopentanoyl chloride) was dissolved in 35 mL of dichloromethane under Ar at 25 °C. Furthermore, 5 equivalents of (3-aminopropyl)triethoxysilane (15.7 g, 70.9 mmol) dissolved in 35 mL of dichloromethane were added dropwise over 30 min at 0 °C and then stirred at 25 °C for 16 h. The resulting solid was washed with diethyl ether to remove dichloromethane using vacuum distillation to produce a white, solid product (2-Azo-initiator). 

#### 2.3.2. Tetra-Functional Initiator (4-Azo-initiator) 

**Scheme 2:**
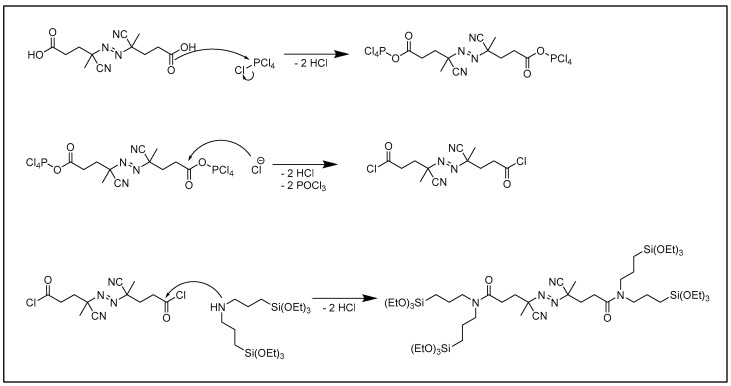
Synthesis of tetrafunctional initiator.

The synthesis of the tetra-functional initiator, substituted on both sides by two pairs of silanes, is shown in Scheme 2. One equivalent of ACVA (5 g, 17.8 mmol) was dissolved in 150 mL of dichloromethane at 25 °C under Ar and then cooled to 0 °C. Then, 2.5 equivalents of phosphorus pentachloride (9.3 g, 44.6 mmol) were added over 30 min, and the mixture was then stirred at 25 °C for 1 h. Using vacuum distillation, 120 mL of dichloromethane was removed using vacuum distillation, 70 mL of hexane was added, and recrystallization at 0 °C produced (E)-4,4′-(diazene-1,2-diyl)bis(4cyanopentanoyl chloride) as a white solid. Then, one equivalent (4.5 g, 14.2 mmol) of the previously obtained (E)-4,4′-(diazene-1,2-diyl)bis(4-cyanopentanoyl chloride) was dissolved in 30 mL of dichloromethane under Ar at 25 °C. Next, 2.1 equivalents (12.7 g, 29.8 mmol) of bis[3-(triethoxysilyl)propyl]amine in 35 mL of dichloromethane were slowly added dropwise at 0 °C, followed by the dropwise addition of four equivalents (5.77 g, 57 mmol) of triethylamine. Then, the mixture was stirred at 25 °C for 14 h. Using vacuum distillation, dichloromethane was evaporated, and the resulting salt was removed with diethyl ether. Finally, n-hexane was added to remove the resulting salt to produce the product (4-Azo-initiator) as an orange liquid. 

### 2.4. Synthesis of Functionalized LqBR 

#### Radical Polymerization 

F-LqBRs were polymerized by radical polymerization in a high-pressure stainless-steel reactor (1L), and the formulation is shown in Table 1. First, the functional initiator (2.183 g of 2-Azo-LqBR or 1.217 g of 4-Azo-LqBR) and THF were filled in the reactor. Then, 60 g of 1,3-butadiene was charged into the reactor using a chamber under an atmosphere of N_2_. The 2-Azo-LqBR and 4-Azo-LqBR were polymerized at 75 °C for 8 h and at 80 °C for 3 h, respectively. The target molecular weight of 4-Azo-LqBR was set considering the molecular weight of the functional groups and the consequent increase in hydrodynamic volume. To achieve this, 4-Azo-LqBR was polymerized at a slightly higher temperature than 2-Azo-LqBR. Subsequently, the mixture was cooled, and the unreacted 1,3-butadiene was discharged using the eject line of the reactor. The solvent was removed through evaporation and precipitated in ethanol to remove residual initiator. As a final step, centrifugation was used to collect the polymers. 

**Scheme 3:**
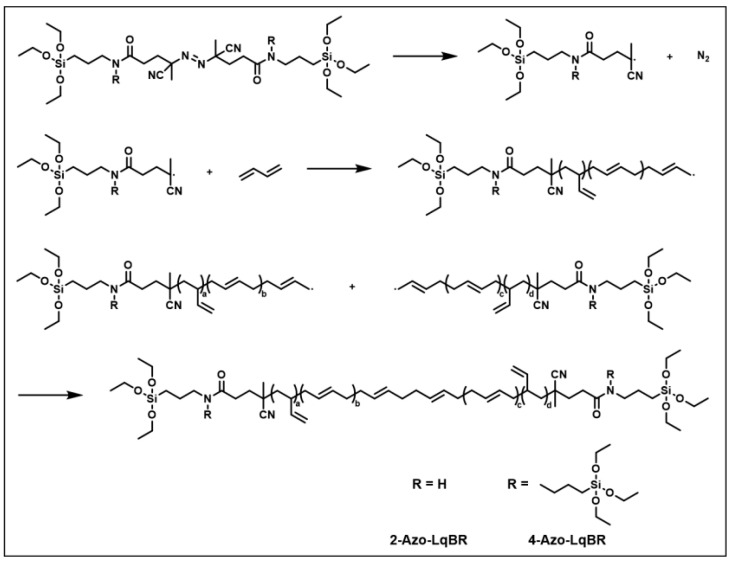
Polymerization of F-LqBRs.

The growing chains can be terminated by coupling or disproportionation in the absence of a chain transfer agent. Since 1,3-butadiene is mainly terminated via coupling [33,34], triethoxysilyl groups were introduced at both ends of a radical initiator. Consequently, silane-terminated polybutadiene was synthesized by using the triethoxysilyl-functionalized initiator (Scheme 3) and the molecular structures of the LqBRs were confirmed using GPC and a ^1^H NMR spectra.

### 2.5. Manufacture of Rubber/Silica Compounds and Vulcanizates

To determine the effect of LqBRs substituted with TDAE oil on the compound’s mechanical and dynamic properties, samples were prepared using the formulations shown in Table 2. In this experiment, the compounds were named according to the processing aid used (TDAE oil, N-LqBR, 2-Azo-LqBR, 4-Azo-LqBR). Compounds were manufactured using an internal kneader (300cc, MIRAESI Company, Gwangju, Republic of Korea) with a fill factor of 0.7, and the three kinds of LqBRs were introduced by replacing 10 phr of the 40 phr of TDAE oil. The kneading process began at 110 °C and continued for 7 min and 40 s. Mixing was then started at 50 °C and continued for 2 min. After each stage of mixing, the compound was sheeted using a two-roll mill. A moving die rheometer was used to measure the optimal cure times of the compounds at 160 °C, and the optimal cure time was used to produce vulcanizates in a press at 160 °C. The detailed process is shown in Table 3.

## 3. Results and Discussion

### 3.1. Synthesis of Functional Initiators

**Figure 1:**
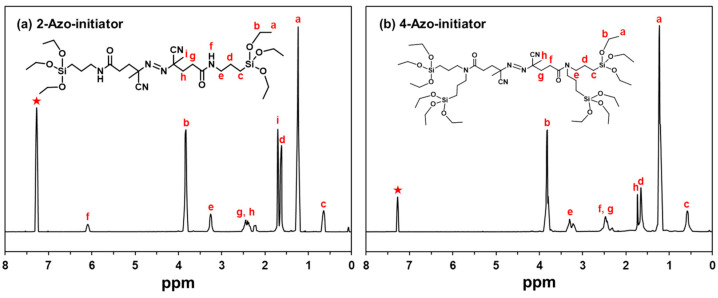
^1^H NMR spectra of the (**a**) 2-Azo-initiator, (**b**) 4-Azo-initiator (★ CDCl_3_ solvent).

The ^1^H NMR peak assignments of the di-functional and tetra-functional initiators are shown in Figure 1a,b, respectively. The chemical shift (δ) was determined using tetramethylsilane as an internal standard, and the following peaks were assigned.

Di-functional initiator: ^1^H NMR (CDCl_3_): δ ppm 0.64 (t, 4H, Si-**CH_2_**-), 1.23 (t, 18H, SiO-CH_2_-**CH_3_**), 1.64 (q, 4H, Si-CH_2_-**CH_2_**), 1.70 (s, 6H, C-**CH_3_**), 2.20–2.52 (m, 8H, CO-CH_2_-**CH_2_,** CO-**CH_2_**), 3.25 (m, 4H, NH-**CH_2_**), 3.84 (q, 12H, SiO-**CH_2_**-), 6.09 (t, 2H, CO-**NH**).

Tetra-functional initiator: ^1^H NMR (CDCl_3_): δ ppm 0.64 (t, 4H, Si-**CH_2_**-), 1.23 (t, 18H, SiO-CH_2_-**CH_3_**), 1.62 (q, 4H, Si-CH_2_-**CH_2_**), 1.70 (s, 6H,C-**CH_3_**), 2.16–2.52 (m, 8H, CO-CH_2_-**CH_2_**, CO-**CH_2_**), 3.52 (m, 4H, N-**CH_2_**), 3.84 (q, 12H, SiO-**CH_2_**-).

### 3.2. Synthesis of F-LqBRs

**Figure 2:**
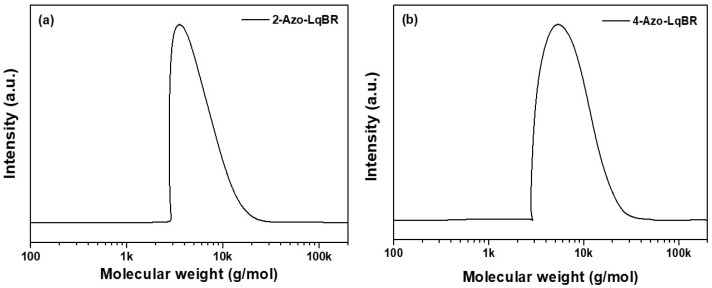
Gel permeation chromatograms of LqBRs: (**a**) 2-Azo-LqBR, (**b**) 4-Azo-LqBR.

**Figure 3:**
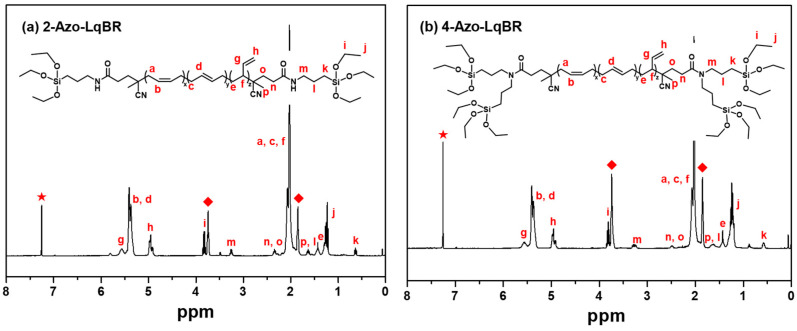
^1^H NMR spectra: (**a**) 2-Azo-LqBR, (**b**) 4-Azo-LqBR (★ CDCl_3_, ◆ THF).

The GPC curves and ^1^H NMR spectra of the F-LqBRs are shown in Figure 2 and Figure 3 and are summarized with the DSC results in Table 4. The GPC measurements showed that the 2-Azo-LqBR and 4-Azo-LqBR have M_n_ values of 4,000 and 5,100 (g/mol) and polydispersity indexes of 1.20 and 1.32, respectively. We regulated the reaction time and temperature conditions according to the target molecular weight, and as a result, the M_n_ and polydispersity index of 4-Azo-LqBR increased compared to 2-Azo-LqBR at the higher temperature. Resonance peaks corresponding to the 1,4-addition (cis, trans), 1,2-addition (vinyl) structure of 1,3-butadiene appear at 5.3–5.5 ppm, 5.5–5.6 ppm, and 4.8–5.0 ppm, respectively. The vinyl contents of the LqBRs were determined according to the proportion of the vinyl peaks to the total 1,3-butadiene peaks. In addition, the chemical shift of the ethoxy groups in alkoxysilane showed a triplet peak at 1.2–1.3 ppm (SiO-CH_2_-**CH_3_**) and a quartet peak at 3.7–3.8 ppm (SiO-**CH_2_**-) [42]. Hence, the functionality of F-LqBRs, which represents the proportion of the chain containing the triethoxysilyl groups to the total number of chains, can be determined by calculating the proportion of the 1,2-vinyl-H integrals in polybutadiene and SiO-**CH_2_**- peak integrals [43]:

(2) $$\frac{S_{\mathrm{Vinyl}\text{-}H}}{S_{\mathrm{Alkoxysilane}\text{-}H}}=\frac{2\;\times \;\left(R_{\mathrm{Vinyl}}\right)\;\times \;\left(\frac{M_{n}}{M_{B}}\right)}{n_{\mathrm{Alkoxysilane}}\;\times \;F}$$

where *S_Vinyl-H_* and *S_Alkoxysilane__-H_* are the peak integrals of 1,2-vinyl-H and alkoxysilane-H, respectively; *R_Vinyl_* is the vinyl content of LqBR; *M_n_* is the number average molecular weight of LqBR; *M_B_* is the molecular weight of 1,3-butadiene; *n_alkoxysilane_* is the hydrogen atom number in alkoxysilane, i.e., -Si-(OC**H_2_**CH_3_)_3_; and *F* is the functionality (triethoxysilyl groups per chain), i.e., “2” means di-functionalized at the ends of macromolecular chains and “4” means tetra-functionalized at the ends of macromolecular chains.

### 3.3. Payne Effect

The Payne effect is used to measure the degree of formation of a filler network. When the filler network is destroyed due to increased strain, the smaller difference (ΔG′) in G′ indicates weaker filler-filler interaction. Using this test, filler dispersion in the rubber matrix can be determined. Furthermore, the hydrodynamic effect, filler-polymer interaction, polymer network, and rubber structure affect the G′ of the compound under high strain where the filler network breaks [44].

**Figure 4:**
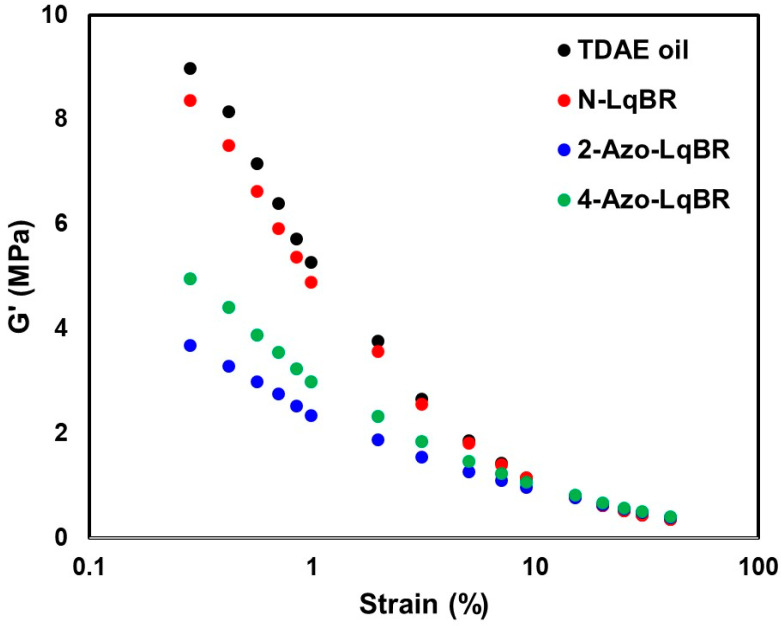
Payne effect in silica masterbatches.

Figure 4 and Table 5 exhibit the Payne effect measurements of the compounds. LqBR exhibited better mixing efficiency, due to its higher viscosity, than that of TDAE oil [19,30]. The addition of LqBR to compounds led to a reduced Payne effect compared to those containing TDAE oil due to improved silica dispersion. In addition, 2-Azo-LqBR and 4-Azo-LqBR have functional groups which can react with silanol groups and hydrophobize the silica surface. Therefore, the compounds with F-LqBR showed significantly lower Payne effect values than the compounds with N-LqBR.

The self-condensation of F-LqBR indicates the formation of a Si-O-Si bond through condensation between alkoxy groups, and there are two types: intramolecular and intermolecular self-condensation. Increasing the number of triethoxysilyl groups in F-LqBR can increase both the reactivity to the filler and to intramolecular and intermolecular self-condensation [45]. Intramolecular condensation can occur within a molecule as the distance between alkoxy groups becomes smaller [46]. The 4-Azo-LqBR compound demonstrated a higher Payne effect than the 2-Azo-LqBR compound. This was due to the higher number of neighboring alkoxy groups within the 4-Azo-LqBR molecule. As a result, the reactivity of 4-Azo-LqBR with silanol groups of the filler is lower than that of 2-Azo-LqBR due to self-condensation. The self-condensation effect in 4-Azo-LqBR was also confirmed by the higher G′ value under high strain (40.04% strain) compared to that of the 2-Azo-LqBR compound.

### 3.4. Cure Characteristics and Mooney Viscosity of the Compounds

Table 6 presents the Mooney viscosity results, a measure of compound processability. The introduction of LqBR enhanced silica dispersion in the rubber matrix and reduced Mooney viscosity compared to the TDAE oil compound. Furthermore, F-LqBR addition resulted in even lower Mooney viscosity values due to the surface hydrophobation of silica that further improved its dispersion in the compound. 4-Azo-LqBR is less reactive with silanol groups than 2-Azo-LqBR due to its self-condensation, which results in a smaller improvement in silica dispersion, and a higher Mooney viscosity compared to that of 2-Azo-LqBR.

**Figure 5:**
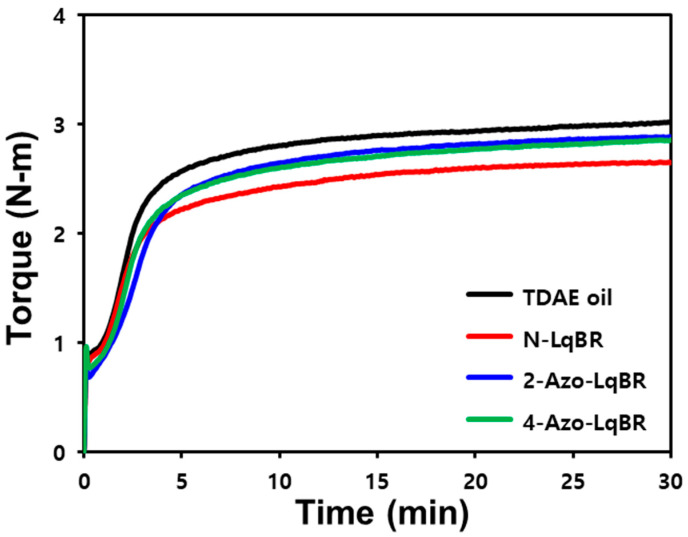
Cure curves of the compounds.

Figure 5 and Table 6 show the cure characteristics obtained using a moving die rheometer. The cure characteristics results exhibit a remarkably low error of less than 1%. The Δtorque (T_max_ − T_min_) value is closely correlated with the crosslink density of a compound [47,48]. The N-LqBR compound exhibited a lower Δtorque value than the TDAE oil compound because N-LqBR consumed the sulfur required for the crosslinking of the base rubber. In contrast, F-LqBRs not only react with silanol groups on the silica surface, but also strengthen filler–rubber interactions by crosslinking with the base rubber. Accordingly, compounds containing the F-LqBRs exhibited higher Δtorque values than those containing N-LqBR. On the other hand, the 4-Azo-LqBR compound exhibited a lower Δtorque than the 2-Azo LqBR compound, which can be attributed to the lower reactivity of the end-functional groups of 4-Azo-LqBR with silanol groups compared to 2-Azo-LqBR.

### 3.5. Solvent Extraction and Crosslink Density

**Figure 6:**
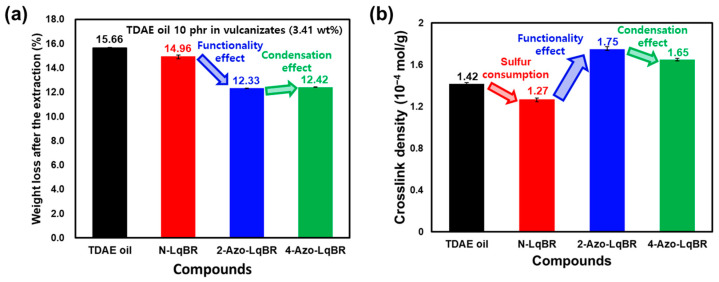
(**a**) Weight loss after solvent extraction and (**b**) crosslink density of the vulcanizates.

Two organic solvents were used to extract organic compounds from vulcanizates, and the amount of extracted organic compounds in the vulcanizates was obtained. First, THF was used to extract the oil and low molecular weight chemicals, followed by n-hexane to extract un-crosslinked LqBR. Figure 6a and Table 7 show the amount of organic compound extracted by the two solvents. The results of solvent extraction exhibit a remarkably low relative standard deviation of 0.06–1.79%. The extraction rate was highest in the TDAE oil compound, which was 15.66 wt%, and it only acts as a plasticizer and does not form chemical bonds in the compound. However, compounds with LqBRs showed lower extraction rates than the TDAE oil compound because LqBRs form a crosslink with the base rubber and bond to the polymer network. Furthermore, the F-LqBR compounds exhibited lower extraction rates compared to those of the N-LqBR compound because they are more strongly bonded to the silica surface by forming chemical bond. However, the 4-Azo-LqBR compound showed a slightly higher extraction rate than the 2-Azo-LqBR compound due to its lower reactivity with silanol groups. Assuming that the 10 phr (3.41 wt%) of TDAE oil was completely extracted and that other additives were equally extracted (30 phr of TDAE oil 10.23 wt% + 2.02 wt%), 79.5% of N-LqBR, 2.2% of 2-Azo-LqBR, and 4.8% of 4-Azo-LqBR were extracted compared to TDAE oil. This indicates that the addition of F-LqBR could prevent migration and maintain the suppleness of the tire to prevent the degradation of its properties.

Table 7 and Figure 6b show the crosslink density of the vulcanizates. The results of crosslink density exhibit a remarkably low relative standard deviation of 0.36–1.88%. The N-LqBR compound exhibited a lower crosslink density than the TDAE oil compound because N-LqBR consumes the sulfur needed for crosslinking with the base rubber instead. However, F-LqBR compounds exhibited a higher crosslink density compared to the TDAE oil compound because of the improved filler–rubber interaction resulting from covalent bonding with silanol groups. The lower crosslink density of the 4-Azo-LqBR compound compared to the 2-Azo-LqBR compound can be attributed to weaker filler–rubber interaction due to self-condensation of 4-Azo-LqBR.

### 3.6. Mechanical Properties and DIN Abrasion Loss

**Figure 7:**
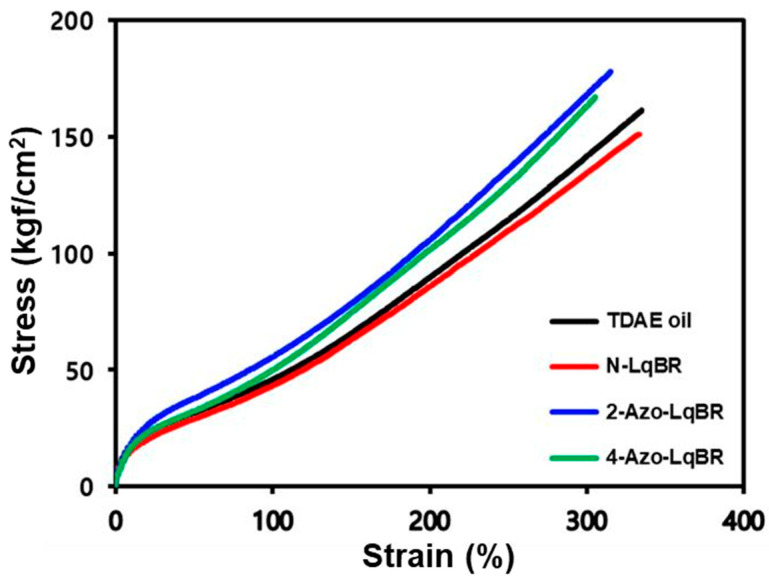
Stress–strain curves of vulcanizates.

The tensile modulus determines rubber stiffness, which is higher for a compound when the crosslink density is high [49]. In Figure 7 and Table 8, the N-LqBR compound exhibited lower M_100_ (modulus at 100% elongation) and M_300_ (modulus at 300% elongation) values than the TDAE oil compound due to lower crosslink density resulting from sulfur consumption of N-LqBR. However, the F-LqBR compounds exhibited higher crosslink density than the TDAE oil compound because of improved filler–rubber interactions resulting from chemical bonding with silanol groups, resulting in higher M_100_ and M_300_ values. In contrast, the 4-Azo-LqBR compound exhibited a lower modulus due to lower filler–rubber interactions compared to 2-Azo-LqBR.

DIN abrasion tests demonstrated that stronger filler–rubber interactions in the compound and lower T_g_ values of the polymer result in the compound having higher abrasion resistance [50,51]. The compounds were manufactured using the same base rubber with the addition of processing aids with different T_g_ values. Thus, the compound with TDAE oil, which demonstrated no chemical reaction and had the highest T_g_ (−50 °C to −44 °C [52]), had the worst abrasion resistance. In contrast, the LqBRs, with lower T_g_ values than TDAE oil, resulted in the compound having superior abrasion resistance. F-LqBRs, which can interact with silanol groups, had higher T_g_ values than the N-LqBR, but the corresponding compounds showed excellent abrasion resistance due to improved filler–rubber interactions. In particular, the compound with 2-Azo-LqBR showed the best abrasion resistance.

### 3.7. Dynamic Viscoelastic Properties

A tire undergoes repeated deformation and recovery when it rotates under the load of a vehicle, resulting in energy loss called hysteresis loss. On icy roads, the tire tread should deform easily, and a large contact area is required for good snow traction [53]. The dynamic viscoelastic properties show that a lower E′ in the low-temperature region results in the tire having better snow traction [54,55]. Tan δ at 60 °C indicates the hysteresis loss and is a measure of the destruction and reformation of the filler network under strain. A higher tan δ at 60 °C is disadvantageous for the rolling resistance of the compound [56]. When the filler is well dispersed and filler–rubber interaction is stronger, it results in a reduced tan δ at 60 °C [19,56].

**Figure 8:**
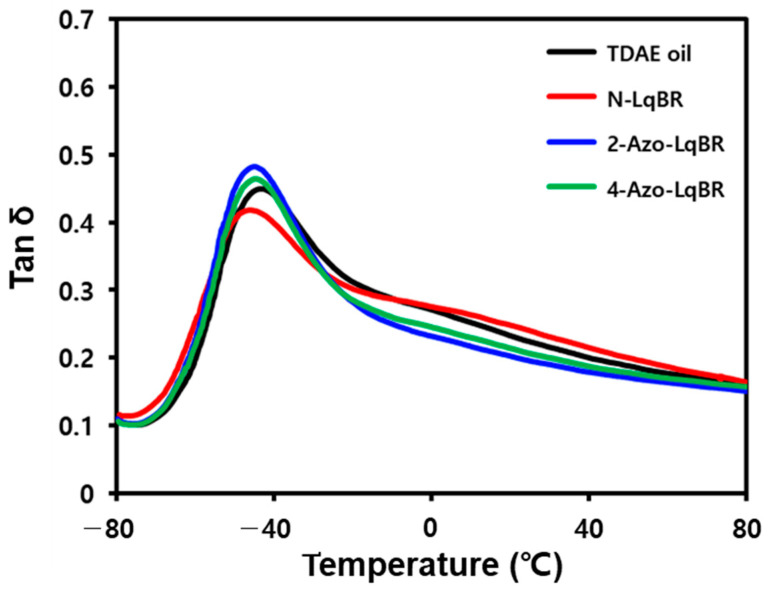
Temperature-dependent tan δ at 0.2% strain curves for the various vulcanizates.

Figure 8 and Table 9 show tan δ values as a function of temperature, measured in our samples using DMA at 0.2% strain. The results indicate that LqBR compounds exhibit lower E′ at −30 °C than the TDAE oil compound. This is due to the effective filler volume fraction being reduced by the improved silica dispersion in the LqBR-containing compounds, as confirmed by the Payne effect results [57]. The N-LqBR compound exhibited a higher tan δ at 60 °C than the TDAE oil compound due to the hysteresis loss caused by the free chain ends of N-LqBR. On the other hand, F-LqBR compounds exhibited lower tan δ at 60 °C values than the TDAE oil compound due to forming chemical bonds between the F-LqBR chain ends and the silica surface. In particular, the 2-Azo-LqBR compound exhibited the lowest tan δ at 60 °C because it had the lowest degree of filler network formation and the strongest bonding to the silica surface due to superior silica hydrophobization compared to the 4-Azo-LqBR compound.

**Figure 9:**
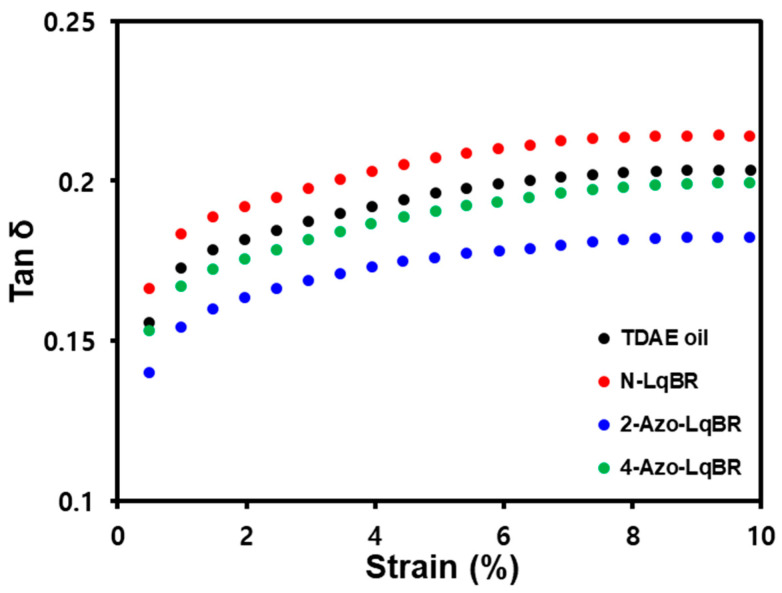
Strain-dependent tan δ at 60 °C curves of the various vulcanizates.

Figure 9 and Table 9 show tan δ values as a function of strain, measured using DMTS at 60 °C. In the high-strain region, the filler network is destroyed to a greater degree than in the low-strain region, resulting in a higher degree of hysteresis [58,59]. Therefore, better silica dispersion results in less filler network formation, thus decreasing the tan δ at 60 °C in the high-strain region. Moreover, in general, compounds with high crosslink density exhibit low tan δ values at 60 °C [48]. Compounds with LqBR formed fewer filler networks due to better silica dispersion compared to the TDAE oil compound. However, the N-LqBR compound exhibited a lower crosslink density due to sulfur consumption, resulting in a higher tan δ at 60 °C compared to the TDAE oil compound. In contrast, the F-LqBR compounds exhibited lower tan δ values at 60 °C in the high-strain region due to a lower hysteresis loss. This is attributed to their excellent silica dispersion, and the 2-Azo-LqBR compound exhibited the lowest tan δ of all samples.

## 4. Conclusions

The objective of this experiment was to investigate the effect of the number of end-functional groups of LqBR used as a processing aid in silica-filled rubber compounds on the compound properties. To achieve this, N-LqBR and F-LqBRs were used as substitutes for TDAE oil to prepare silica-filled rubber compounds, and the properties were evaluated.

The experimental results showed that LqBR acts as a processing aid and forms crosslinks with the base rubber, improving migration resistance. In particular, 2-Azo-LqBR and 4-Azo-LqBR with functional groups formed chemical bonds with the silica surface, showing better silica dispersion for N-LqBR. However, the 4-Azo-LqBR compound, with an end-functionality of four, demonstrated poor silica dispersion compared to the 2-Azo-LqBR compound due to increased self-condensation.

As a result of the sulfur consumption of LqBR, the crosslink density of the N-LqBR compound was lower compared to that of the TDAE oil compound. Despite the sulfur consumption, 2-Azo-LqBR and 4-Azo-LqBR showed a strong interaction between the filler and base rubber due to the functional groups, resulting in higher crosslink density than that of the TDAE oil compound. Crosslink density had a strong correlation with the mechanical properties of the compounds. The T_g_ of the processing aids and the strength of the filler–rubber interaction determined the abrasion resistance. The dynamic viscoelastic properties showed that improvements in the snow traction and rolling resistance of the compound could be expected by applying the developed F-LqBRs. Rolling resistance is mainly dependent on hysteresis loss due to the free chain end of the polymer, the filler network destruction and reformation, and the filler–rubber interaction. Consequently, the compound with 2-Azo-LqBR, which had the strongest reactivity with the silica surface, exhibited the lowest tan δ at 60 °C because of the improved silica dispersion and filler–rubber interaction. Accordingly, 2-Azo-LqBR showed the best performance among the studied processing aids, and the optimized end functionality of triethoxysilyl group of F-LqBR is two.

This study was novel in that we controlled the end functionality of telechelic polymer, which requires a high level of technics, and confirmed the effect of reactivity according to the number of end-functional groups of LqBR on the physical properties of the tire tread compound. The results obtained in this study suggest the guideline of designing F-LqBR structures that concurrently improve fuel efficiency and wear resistance for electric vehicles that require improved performances and performance sustainment and prevent deterioration of physical properties over time.

## Figures and Tables

**Table 1:** Formulation of F-LqBRs (unit: g).

	Organic Compounds	2-Azo-LqBR	4-Azo-LqBR
Di-functional initiator	2-Azo-initiator	2.183	-
Tetra-functional initiator	4-Azo-initiator	-	1.217
Monomer	1,3-butadiene	60	60
Solvent	THF	420	300

**Table 2:** Formulation of compounds (unit: phr).

Sample	TDAE Oil	N-LqBR	2-Azo-LqBR	4-Azo-LqBR
SSBR	80	80	80	80
BR	20	20	20	20
Silica	120	120	120	120
X50S	20	20	20	20
DPG	2	2	2	2
TDAE oil	40	30	30	30
N-LqBR	-	10	-	-
2-Azo-LqBR	-	-	10	-
4-Azo-LqBR	-	-	-	10
Wax	1	1	1	1
TMQ	1	1	1	1
ZnO	3	3	3	3
Stearic acid	1	1	1	1
6PPD	2	2	2	2
Sulfur	1.3	1.3	1.3	1.3
CBS	1.6	1.6	1.6	1.6
ZBEC	0.1	0.1	0.1	0.1

Notes: phr, parts per hundred rubber.

**Table 3:** Mixing procedures.

	Time, mins	RPM	Action
Silica masterbatch (SMB) mixing	00:00–00:40	20	Add rubber (initial temp.: 110 °C)
	00:40–01:40	40	Add silica ^1^/_2_, X50S ^1^/_2_, DPG ^1^/_2_, processing aid ^1^/_2_
	01:40–02:40	40	Add silica ^1^/_2_, X50S ^1^/_2_, DPG ^1^/_2_, processing aid ^1^/_2_
	02:40–05:00	60	Add additives
	05:00–05:30	60	Ram up
	05:30–07:40	50	Extra mixing and dump (dump temp.: 150–155 °C)
Final masterbatch (FMB) mixing	00:00–00:20	20	Add SMB (initial temp.: 50 °C)
	00:20–02:00	40	Add sulfur, CBS, ZBEC and dump (dump temp.: 80–90 °C)

**Table 4:** Characteristics of LqBRs.

Property	Unit	N-LqBR	2-Azo-LqBR	4-Azo-LqBR
Sample M_n_	g/mol	4400	4000	5100
Polydispersity index (Đ)	-	1.04	1.20	1.32
Vinyl content (% in BD)	-	15	19	19
T_g_	°C	−95	−86	−78
End functionality (Si per chain)	-	0	2.3	4.3

**Table 5:** G′ values (MPa) of compounds.

Property	TDAE Oil	N-LqBR	2-Azo-LqBR	4-Azo-LqBR
G′ (at 0.28% strain)	8.98	8.37	3.69	4.96
G′ (at 40.04% strain)	0.36	0.36	0.40	0.41
ΔG′ (at 0.28–40.04% strain)	8.62	8.01	3.29	4.55

**Table 6:** Cure characteristics and Mooney viscosities of the compounds.

Property	Unit	TDAE Oil	N-LqBR	2-Azo-LqBR	4-Azo-LqBR
Mooney viscosity (ML_1+4_ at 100 °C, FMB)	MU	153	147	116	119
T_min_	N-m	0.88	0.82	0.68	0.77
T_max_	N-m	3.02	2.65	2.88	2.85
Δtorque	N-m	2.14	1.83	2.20	2.09
t_10_	min:sec	1:11	1:05	1:09	1:15
t_90_	min:sec	10:02	11:55	10:40	11:51

**Table 7:** Weight loss after solvent extraction and crosslink density of the vulcanizates.

Property	Unit	TDAE Oil	N-LqBR	2-Azo-LqBR	4-Azo-LqBR
Weight loss after extraction	%	15.66	14.96	12.33	12.42
Weight loss after extraction in 10 phr of oil and LqBR	%	100	79.5	2.2	4.8
Crosslink density	10^−4^ mol/g	1.42	1.27	1.75	1.65

**Table 8:** Mechanical properties and abrasion resistance of vulcanizates.

Property	Unit	TDAE Oil	N-LqBR	2-Azo-LqBR	4-Azo-LqBR
M_100_	kgf/cm^2^	46	43	55	50
M_300_	kgf/cm^2^	142	135	168	163
Elongation at break	%	335	334	315	305
Tensile strength	kgf/cm^2^	161	151	178	167
DIN abrasion loss	mg	109	96	91	93

**Table 9:** Viscoelastic properties of the vulcanizates.

Property	Unit	TDAE Oil	N-LqBR	2-Azo- LqBR	4-Azo- LqBR
Number end functional groups	-	N/A	0	2	4
T_g_	°C	−43.1	−45.9	−44.6	−44.8
E′ at −30 °C	MPa	164	163	137	152
Tan δ at 60 °C (0.2% strain, temperature sweep)	-	0.177	0.188	0.165	0.170
Tan δ at 60 °C (5% strain, strain sweep)	-	0.196	0.207	0.176	0.190

## Data Availability

Data presented in this study are available upon request from the corresponding author.
